# Checkpoint Inhibitor-Related Capillary Leak Syndrome (CLS)

**DOI:** 10.7759/cureus.55719

**Published:** 2024-03-07

**Authors:** Ahmad Raja, Amrat Kumar, Muhammad Abdullah, Muhammad Daniyal, Anamm Polani

**Affiliations:** 1 Internal Medicine, Bassett Medical Center, Cooperstown, USA; 2 College of Medicine, Islamic International Medical College, Lahore, PAK

**Keywords:** immune checkpoint inhibitors, immunotherapy, generalized anasarca, systemic capillary leak syndrome, immune-related adverse event (irae)

## Abstract

Immune checkpoint inhibitors (ICIs) are now being widely used for the treatment of various malignancies, but they have a distinctive set of side effects due to the overactivation of the immune system, which is important to recognize. Capillary leak syndrome (CLS) is a rare but potentially life-threatening side effect of ICIs that causes a significant increase in the permeability of capillaries, leading to the leakage of plasma-containing proteins from these small vessels. This condition results in several clinical features, including edema, hypotension, hypoalbuminemia, and hemoconcentration. Timely recognition and discontinuation of the offending immunotherapy can optimize outcomes. Treatment is focused on supportive care and prompt initiation of immunosuppressants, such as steroids.

## Introduction

We present a case of a 47-year-old female with a medical history of stage III vaginal melanoma treated with radiation and combination immunotherapy (nivolumab/ipilimumab) who presented to the hospital with nausea, fatigue, anasarca, and weight gain. She also had a previous history of severe colitis caused by immunotherapy requiring hospitalization and treatment with steroids. The examination was remarkable for hypotension and generalized anasarca. Workup was significant for severe hypoalbuminemia without proteinuria or evidence of kidney injury. She also had normal liver function tests and an unremarkable transthoracic echocardiogram (TTE) ruling out common causes of anasarca, like nephrotic syndrome, liver disease, or congestive heart failure. Steroids were started due to a high clinical suspicion of capillary leak syndrome (CLS), and she responded well with significant improvement in edema, blood pressure, and generalized strength and was eventually discharged home with a slow taper of steroids and oral diuretics.

## Case presentation

We present the case of a 47-year-old female who had previously been diagnosed with stage III vaginal melanoma that had extended to the distal urethra and left inguinal lymphadenopathy. Due to the extent of her disease, she was determined not to be a candidate for surgery and underwent neoadjuvant pelvic external beam radiation therapy, followed by high-dose-rate (HDR) brachytherapy. In addition, she received combination therapy of nivolumab and ipilimumab while waiting for the results of molecular testing for the BRAF gene, which ultimately returned negative for the mutation. However, after only two cycles, her immunotherapy was interrupted due to severe immune-mediated colitis that required hospitalization for intravenous (IV) hydration and immunosuppression with high-dose steroids. Although she initially responded well to treatment and was discharged on a slow steroid taper, she abruptly discontinued therapy without medical advice three weeks later after her diarrhea had subsided.

Over the following weeks, the patient experienced weight gain, progressive lower extremity edema, nausea, poor appetite, and fatigue. She gained approximately 20 pounds of weight in eight weeks and reported difficulty ambulating due to the heaviness in her legs. In addition, she experienced exertional dyspnea but did not have orthopnea or paroxysmal nocturnal dyspnea (PND). Upon being sent to the hospital for further evaluation, she presented with dry mucous membranes and lethargy, although she remained alert and oriented without apparent distress. Her blood pressure was low (82/57) with a normal heart rate of 68, but all other vital signs were normal (respiratory rate 14, temperature 36.7 degrees Celsius, and saturation 94% on room air). She did not have jugular venous distention and her chest was clear upon auscultation bilaterally. The patient exhibited diffuse pitting edema up to her groin bilaterally, which extended up to the abdominal wall and sacrum, although no obvious edema was noted in the upper extremities. Her weight had increased from 145 pounds (lbs) two months prior to 163 lbs at the time of presentation.

Investigations

On admission, the patient's blood workup was remarkable for hypokalemia (3.0 mmol/L), hypomagnesemia (1.7 mg/dL), severe hypoalbuminemia (1.9 g/dL), hypoproteinemia (total protein 3.7 g/dL), and chronic anemia (hemoglobin 9.2 g/dL, hematocrit 28%). However, other electrolytes, liver function tests (LFTs), blood urea nitrogen (BUN), serum creatinine (Cr), lactic acid levels, white blood cell count (WBC), and platelets (Plt) were within normal ranges. Table 1 shows the laboratory workup done on day 1, day 2, and day 3.

**Table 1:** Laboratory workup done on day 1, day 2, and day 3 BNP: brain natriuretic peptide, CO_2_: bicarbonate, BUN: blood urea nitrogen, ALT: alanine transaminase, AST: aspartate aminotransferase, MCV: mean corpuscular volume, MCH: mean corpuscular hemoglobin, MCHC: mean corpuscular hemoglobin concentration

Investigations	Day 1	Day 2	Day 3	Reference ranges	Units
Troponin	<0.3			<= 15	pg/ml
BNP	87			6-100	pg/mL
Sodium	143	142	142	136-145	mmol/l
Potassium	3	2.8	4	3.5-5.1	mmol/l
Chloride	106	107	105	98-110	mmol/l
Glucose	71	107	151	70-139	mg/dl
CO2	24	25	23	21-31	mmol/l
Anion gap	13	11	14	6-14	mmol/l
BUN	10	7	5	7-25	mg/dl
Creatinine	0.7	0.7	0.7	0.7-1.3	mg/dl
ALT	18		12	7-72	U/L
AST	20		18	13-39	U/L
Alkaline phosphatase	107		77	34-104	U/L
Total bilirubin	0.3		0.5	0.3-1.0	mg/dl
Albumin	1.9	2.7	3.7	3.5-5.7	g/dL
Total protein	3.7	3.7	5.9	6-8.3	g/dL
White blood cells	6.1			3.7-10.6	x 10E^3^ cells/uL
Hemoglobin	9.2			11.5-15.5.0	g/dl
Hematocrit	28			34.0-46.0	%
MCV	100.4			81.0-99.0	fL
MCH	33.5			27-33.5	pg
MCHC	32.2			31.5-35.5	g/dl
Platelets	275			140-425	x 10E^3^ cells/uL
Lactic acid	0.7			0.5-2.2	mmol/L

A chest X-ray (CXR) was normal with no evidence of pulmonary edema or infiltrates. The patient underwent a CT scan of the abdomen and pelvis, which revealed diffuse mesenteric edema with minimal ascites and diffuse soft tissue anasarca but was otherwise unremarkable. Brain natriuretic peptide (BNP) levels were within normal range. Transthoracic echocardiogram (TTE) was unremarkable with a normal ejection fraction and diastolic function. Bilateral lower extremity venous duplex scans were negative for deep venous thrombosis (DVT). The patient also had normal thyroid function and morning cortisol levels. Urinalysis, urine protein, and microalbumin/Cr ratio were all within normal limits.

Differential diagnosis

Upon evaluating the patient's medical history and symptoms, we considered several potential diagnoses. While sepsis was initially a concern, the absence of leukocytosis, fever, or localizing signs of infection, along with the gradual onset of symptoms over several weeks, made this diagnosis less likely. A CXR and urinalysis did not reveal any significant abnormalities, and subsequent urine and blood cultures also came back negative for infection. Nephrotic syndrome, characterized by severe hypoalbuminemia and anasarca, was another possibility, but the patient's normal kidney function and lack of proteinuria ruled out this diagnosis. Similarly, tests for congestive heart failure and liver disease, including BNP, transthoracic echocardiogram (TTE), and LFTs, were all within normal range, making these conditions unlikely. Although the patient had recently received high-dose steroids and was experiencing hypotension, fatigue, and other symptoms that could suggest adrenal insufficiency, normal morning cortisol levels made this diagnosis unlikely. Finally, while bilateral DVT was a concern given the patient's known malignancy and massive lower extremity edema, a venous duplex ruled out this diagnosis.

Treatment

Initially, the patient was carefully resuscitated using IV albumin to maintain her mean arterial pressure (MAP) above 65, while monitoring for pulmonary edema and compartment syndrome. Fortunately, she did not require any vasopressors, and diuresis was not feasible due to low blood pressure. The medical team strongly suspected CLS based on her history of immune-mediated colitis, and other major causes of anasarca and hypotension were ruled out. Consequently, she was started on a 1 mg/kg (70 mg daily) dose of prednisone, which was gradually tapered to 50 mg daily by the time of her discharge in six days. Once her blood pressure had improved, IV fluids were discontinued, and diuretics were gradually introduced. Dietary protein supplements were started to improve overall nutritional status, and she began to increase mobility with physical therapy. Compression stockings were also fitted to alleviate the edema. She was discharged on a slow steroid taper over six weeks and advised to follow up with oncology and her primary care physician (PCP).

Outcome and follow-up

The patient exhibited an exceptional response to steroid therapy, as evidenced by the restoration of her blood pressure to normal levels within a single day, obviating the requirement for IV fluid administration. Furthermore, her serum albumin levels also revealed a notable improvement, increasing from 1.9 g/dL to 2.7 g/dL within 24 hours, and further rising to 3.7 g/dL by the 48-hour mark. On examination, her edema steadily improved each day and was limited to below the knees by the time of her discharge after six days. Notably, her weight had decreased from 163 lbs on admission to 151 lbs at the time of discharge. Two weeks later, during a follow-up visit at the oncology clinic, her peripheral edema had further improved and was now limited to her ankles bilaterally. In addition, her weight had decreased to 141 lbs, and her blood pressure and albumin levels had both improved.

Despite these positive developments, the patient's melanoma presented a challenge, as she was no longer a candidate for immune checkpoint inhibitors (ICI) therapy and her cancer was BRAF negative, limiting treatment options. As such, she was referred to a melanoma specialist for a second opinion and consideration of chemotherapy.

## Discussion

ICIs have revolutionized cancer treatment by enhancing the antitumor immune response by targeting programmed death 1 (PD-1) and its ligand (PD-L1) or cytotoxic T-lymphocyte antigen-4 (CTLA-4) [1]. However, these drugs can also trigger an autoimmune response, causing immune-related adverse events (irAEs), including CLS. CLS is a rare medical condition characterized by an increase in capillary permeability, resulting in the leakage of plasma-containing proteins from the vessels. It manifests with several clinical features, including edema, hypotension, hypoalbuminemia, and hemoconcentration. The precise understanding of the pathophysiology behind CLS induced by ICIs remains incomplete. Nonetheless, research indicates that pivotal factors like vascular endothelial growth factor, sphingosine-1-phosphate, and cytokines, including interleukin-2 and tumor necrosis factor-alpha, are believed to be influential. The involvement of the endothelial glycocalyx layer disruption plays a significant role in the pathophysiological mechanisms of CLS, leading to increased vascular permeability and fluid leakage [2,3].

Cancer and immunotherapy have been reported to be significant contributing factors for CLS, which can occur secondary to autoimmune disorders, infections, or drug usage [4]. CLS can be challenging to diagnose as its clinical presentation can mimic other illnesses, such as sepsis or allergy. Laboratory tests, such as hypoalbuminemia, hemoconcentration, and proteinuria, may help in diagnosis but are not always reliable [2]. Studies have shown that several ICIs, including nivolumab, pembrolizumab, and ipilimumab, have been associated with CLS [5]. The severity of CLS symptoms and the organs affected determine its management. The first approach is to stop the ICI therapy as soon as symptoms are identified. Supportive care for patients with moderate to severe CLS may include oxygen therapy, IV fluids, and vasopressor support. High-dose corticosteroids (1-2 mg/kg/day) are recommended for patients with moderate to severe symptoms, while immunosuppressive treatments, such as intravenous immunoglobulin (IVIG) or plasma exchange, may be considered for those with life-threatening symptoms [6,7]. The underlying etiology, the severity of the symptoms, and how quickly CLS is diagnosed and treated all affect the prognosis. A systematic review and meta-analysis of drug-induced CLS in cancer patients found that the incidence of the condition varied greatly based on the type and dosage of drugs used, with a possible incidence of up to 16.8%. Individuals with pre-existing comorbidities were found to be at higher risk of mortality and morbidity after developing CLS [8].

The literature describes three case reports of patients who developed CLS as a result of ICI therapy. The first case involves a 69-year-old female with metastatic melanoma who developed severe CLS after six months of pembrolizumab treatment. The second case features a 73-year-old male with non-small cell lung cancer who developed CLS following nivolumab and ipilimumab therapy. The third case involves a 52-year-old female with metastatic melanoma who developed nivolumab-induced CLS. In all cases, prompt treatment with high-dose corticosteroids and/or IVIG resulted in significant improvement in symptoms and laboratory values [9-11].

To manage and detect these potentially fatal irAEs, healthcare personnel should be familiar with the latest management guidelines, including those for CLS. The American Society of Clinical Oncology (ASCO) and the Society for Immunotherapy of Cancer (SITC) have published clinical practice guidelines for the management of irAEs, including CLS [6,7]. In conclusion, although ICIs have dramatically improved cancer treatment, they may also result in irAEs, including CLS, which can be life-threatening. Early diagnosis and prompt treatment are crucial for a favorable prognosis. Healthcare providers need to be aware of these uncommon but serious adverse events and maintain a high index of suspicion to manage and detect them promptly.

## Conclusions

ICIs have a distinctive set of side effects due to the overactivation of the immune system, which is important to recognize. Patients receiving ICIs should be closely monitored for adverse effects, including those affecting the gastrointestinal tract, liver, endocrine system, lungs, and skin. CLS is a rare but potentially life-threatening side effect of ICIs that causes a significant increase in the permeability of capillaries, leading to the leakage of plasma-containing proteins from these small vessels. It can cause several clinical features, including edema, hypotension, hypoalbuminemia, and hemoconcentration. The differential diagnosis of anasarca should include nephrotic syndrome, liver disease, congestive heart failure, and CLS in patients receiving ICIs. Timely recognition and discontinuation of the offending immunotherapy can optimize outcomes in patients with CLS. Treatment is focused on supportive care and prompt initiation of immunosuppressants, such as steroids.
